# Random sorting of *Campylobacter jejuni* phase variants due to a narrow bottleneck during colonization of broiler chickens

**DOI:** 10.1099/mic.0.000669

**Published:** 2018-06-01

**Authors:** Joseph J. Wanford, Lea Lango-Scholey, Harald Nothaft, Yue Hu, Christine M. Szymanski, Christopher D. Bayliss

**Affiliations:** ^1^​ Department of Genetics and Genome Biology, University of Leicester, Leicester, UK; ^2^​ Department of Biological Sciences, University of Alberta, Edmonton, Canada; ^3^​ Department of Microbiology and Complex Carbohydrate Research Center, University of Georgia, Athens, USA

**Keywords:** *Campylobacter*, broiler chickens, phase variation, population bottleneck

## Abstract

Phase variation (PV), involving stochastic switches in gene expression, is exploited by the human pathogen *Campylobacter jejuni* to adapt to different environmental and host niches. Phase-variable genes of *C. jejuni* modulate expression of multiple surface determinants, and hence may influence host colonization. Population bottlenecks can rapidly remove the diversity generated by PV, and strict single-cell bottlenecks can lead to propagation of PV states with highly divergent phenotypes. Using a combination of high-throughput fragment size analysis and comparison with *in vivo* and *in silico* bottleneck models, we have characterized a narrow population bottleneck during the experimental colonization of broiler chickens with *C. jejuni* strain 81-176. We identified high levels of variation in five PV genes in the inoculum, and subsequently, massively decreased population diversity following colonization. Each bird contained a dominant five-gene phasotype that was present in the inoculum indicative of random sorting through a narrow, non-selective bottleneck during colonization. These results are evidence of the potential for confounding effects of PV on *in vivo* studies of *Campylobacter* colonization factors and poultry vaccine studies. Our results are also an argument for population bottlenecks as mediators of stochastic variability in the propensity to survive through the food chain and cause clinical human disease.

## Introduction

*Campylobacter jejuni*, a Gram-negative, spiral-shaped bacterium, is a common commensal of chickens and is usually associated with an asymptomatic colonization phenotype [1]. Disease in humans ensues following ingestion of undercooked poultry products and often manifests as a prolonged, but self-limiting, bloody diarrhoea [2]. This bacterial species is the main cause of gastroenteritis in the developing world [3], with these conditions occasionally progressing to the auto-immune disease Guillain–Barré Syndrome, a flaccid, full-body paralysis. Despite a clear relationship between colonization of poultry and human disease, we still have a very limited understanding of the bacterium’s mechanisms of adaptation to the chicken host, and the changes in phenotypic heterogeneity following spread through a poultry population.

Phase variation (PV) is stochastic, reversible switching of gene expression, which allows bacteria to respond to fluctuating environmental and within-host selection pressures [4]. In *C. jejuni,* PV is mediated by the presence of homopolymeric, simple-sequence repeats (SSRs) within coding regions of the genome [5]. Insertions and deletions of single nucleotides in these regions, through slippage of the replicative polymerase, can lead to frameshift mutations resulting in the switching of genes from an ON to an OFF state (coded 1 and 0, respectively), and vice versa [6]. *C. jejuni* is known to encode ~30 phase-variable genes per genome [7], giving rise to a potential for ~2^30^ differing expression states – termed phasotypes. As an example, a theoretical bacterium with four phase-variable genes, with the first gene switched ON and the other genes switched OFF, would have a phasotype coded as 1-0-0-0.

Bacteria are known to undergo population bottlenecks during transmission between hosts and migration between different host compartments [8–10]. These bottlenecks are thought to vary in size from single cells to large populations of a thousand or more cells. The rapid nature of diversification afforded by SSR-mediated phase variable gene expression can mitigate the reduction in population diversity imposed by small bottlenecks. Recently, we have utilized both simulations and experimental models to show that single-cell bottlenecks produce significant reductions in population diversity, whilst bottlenecks of larger sizes carry forward higher amounts of diversity [11]. As many PV genes of *C. jejuni* are known virulence determinants [12, 13], we have proposed that bottlenecks imposed on phase-variable populations have the potential to alter disease outcome.

Population bottlenecks are likely to occur and to impact on PV dynamics when poultry are exposed to *C. jejuni* populations. We previously investigated the PV status of multiple phase-variable genes in *C. jejuni* populations isolated from naïve and non-responder N-glycan-vaccinated broiler chickens, after experimental challenge [14]. PV states exhibited similar profiles in populations from both groups of chickens, indicating that PV did not facilitate escape of vaccine responses. We describe herein an in-depth analysis of PV in these populations. We observed evidence of a serendipitous population bottleneck that is likely to have occurred during colonization of broiler chickens after inoculation with a *C. jejuni* population harbouring multiple phasotypes. We propose that the inocula phasotypes were subject to random sorting during passage through a colonization-associated single cell bottleneck, and discuss the implications for the acquisition of disease-causing populations of *C. jejuni* in humans.

## Methods

### Infection of broiler chickens and sample preparation

**Fig. 1.**
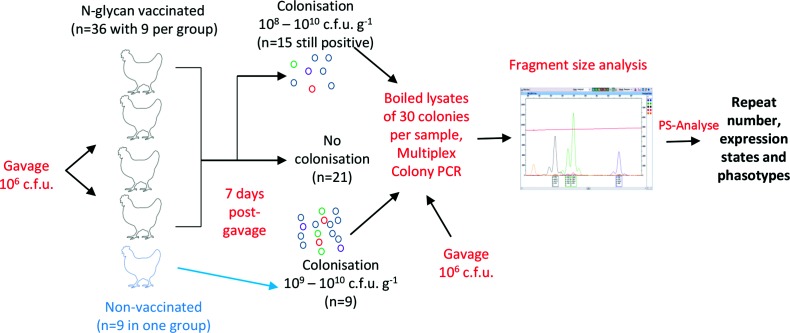
Sample source and workflow for PV analysis of single colonies from vaccinated and non-vaccinated chickens. Four groups of nine birds, vaccinated on days 7 and 21 with an *E. coli* strain expressing the *C. jejuni* N-glycan with and without co-inoculation of probiotics, and one group of non-vaccinated birds were orally gavaged with *C. jejuni* strain 81-176 on day 28. At 7 days post-challenge, *C. jejuni* colonization levels were assessed by serial dilution of caecal contents on selective media. For samples exhibiting colonization, boiled lysates were prepared from 30 colonies and these lysates were then analysed by multiplex PCR, and high-throughput fragment size analysis of 19 polyG tracts. Fragments sizes were converted into repeat numbers and expression states using PS-Analyse followed by derivation of phasotypes.

Samples were derived from an *in vivo* experiment to test the efficacy of the N-glycan-based *Campylobacter* vaccine as described in Nothaft *et al*. [14] and outlined in Fig. 1. Individually housed chickens were either mock-treated with PBS (negative control group) or challenged with 1×10^6^ c.f.u. of *C. jejuni* strain 81–176 by oral gavage at day 28 (positive control group, bird numbers 2–10). Chickens in the vaccine groups were immunized at 7 and 21 days of age prior to challenge at day 28 (birds 11–25). Caecal samples were collected on day 35 and serial dilutions were plated on *Campylobacter*-selective Karmali agar. The samples consisted of boiled lysates of 30 individual colonies derived from serial dilutions of the gavage (i.e. inoculum given at day 28) and from single colonies obtained after serial dilutions of the chicken caecal contents (30 colonies per bird) obtained on day 35. A sample size of 30 colonies was selected, as this provides accurate measurement of diversity statistics for 32–128 phasotypes [11]. All experiments were carried out in accordance with the protocol approved by the Animal Care and Use Committee at the University of Alberta.

### Identification of PV genes in *C. jejuni* strain 81-176

The genome sequence for strain 81-176 was analysed with Phasome*It* (Aidley *et al.*, in preparation), using strain NCTC11168 as a reference, to identify all putative PV loci. Subsequent sequence analyses were performed using Artemis, A Plasmid Editor (APE) and blast. The dataset used to identify PV genes in *C. jejuni* strain 81-176 can be accessed online at: http://www.jackaidley.co.uk/phasome/campy/.

### Sequencing of repeat tracts of *C. jejuni* strain 81-176 in a control DNA sample

Genomic DNA was prepared from a culture of *C. jejuni* strain 81-176 grown overnight on Mueller–Hinton agar plates at 37 °C under micro-aerobic conditions (5 % O_2_, 10 % CO_2_, 85 % N_2_). Repeat tracts of identified PV genes were amplified using primers spanning the repetitive regions as described by Nothaft *et al.* [14]. Sanger sequencing was performed using forward and reverse primers by GATC Biotech, UK. Chromatograms were analysed using FinchTv to determine repeat numbers.

### Multiplex PCR amplification and fragment analysis of PV loci in experimental *C. jejuni* populations

Repeat numbers for the phase-variable genes of *C. jejuni* strain 81-176 were determined using a modification of our established multiplex PCR assay [15] and as described [14]. Briefly, SSRs of PV genes were amplified using four multiplex PCR reactions and analysed in a combined 19-gene GeneScan assay [14]. Multiplex PCR groups were designed to contain amplicons from different regions of the genome to avoid competition or interference during amplification. A control DNA sample (see above) was included on each GeneScan plate in order to calibrate fragment size with a known repeat number as a control for variability in each autosequencer run. PCR amplification was performed using a fluorescent forward primer, and a non-fluorescent reverse primer, on single colony lysates in a 96-well PCR plate. In total, DNA from 30 colonies from each experimental group was analysed. PCR reactions were performed with Bioline Taq Polymerase according to the manufacturer’s instructions. PCR conditions were used as previously described [15]. Subsequently 2.5 µl of each PCR product were pooled together and subjected to A-tailing as previously described [14]. A 1.5 µl sample of each A-tailed PCR mix was then added to 9.25 µl formamide and 0.25 µl GS600LIZ ladder (Thermo Fisher Scientific). Samples were then subjected to Genescan fragment size analysis on an ABI3300 autosequencer.

### High-throughput determination of repeat number, and assigning of ON/OFF states

Genescan data files were analysed in Peakscanner (Applied Biosystems), and data were exported as a combined table (.csv file). Data were subsequently analysed using PSAnalyse, as previously described, to determine the repeat numbers of PV genes for each colony, and their ON/OFF states [15]. Calibration files were developed using repeat tract length data obtained from Sanger sequencing of each PV locus of a control sample (see above), and a peakset file was derived from Genescan data arising from PCR amplification of this control sample. The output of PSAnalyse is a series of Excel files containing tract lengths and expression states for each locus. Subsequent analyses were performed in Microsoft Excel and Graphpad Prism. A diagrammatic representation of this experimental workflow is shown in Fig. 1.

### Diversity and divergence calculations

Phasotype diversity and divergence were calculated using previously described equations [11].

Briefly, phasotype diversity was quantified using Shannon Equitability and the following equation:

$$Diversity=\frac{S}{Smax}=-\frac{1}{G}\sum plog2p$$

where G is the number of genes making up a phasotype, and *p* is the phasotype frequency in the population. The maximum diversity is given as 1 (equal mixture of all possible phasotypes), and the minimum diversity is 0 (whole population expressing a single phasotype).

Divergence was quantified using population separation, using the following equation:

Divergence=1−∑{p,q}

where *p* and *q* are the phasotype proportions for each population. The maximum divergence is 1 (no phasotype in common), and the minimum divergence is 0 (identical types and proportions of phasotypes).

### *In silico* imposition of bottlenecks on phase-variable populations

Population bottlenecks were simulated using our previously published script [11]. This simplified model assumes uniform doubling times and phase-variable genes with symmetrical switching rates (i.e. identical probabilities for ON-to-OFF and OFF-to-ON switching) of 1 in 500 mutations/division at each binary division. Bottlenecks, as indicated, were applied when each population reached its maximum size (~10^9^ c.f.u.), and for runs with multiple imposed bottlenecks (Figs S1–S3, available in the online version of this article) these output populations were used to seed the next run of the simulator. Modelling was carried out in Python 3.3 (http://www.python.org) with use of NumPy 1.9.3 to generate random numbers. The parameters of the model were altered to account for a theoretical population of bacterial cells with five PV genes, as present in the *in vivo* dataset.

The population bottleneck simulator is available from Dryad (https://doi.org/10.5061/dryad.p46n0).

### Statistical comparison of *in vivo* data to simulated botttlenecks

Similarities in the diversity and divergence scores between *in vivo* and simulated output populations were assessed with a non-parametric Permutational Multivariate Analysis of Variance (PERMANOVA) test [16]. Statistical analyses were performed in R.

## Results

### The *C*. *jejuni* strain 81-176 genome contains 20 functionally diverse PV genes

Firstly, we used Phasome*It –* a novel programme which identifies PV genes through association with simple sequence repeats in the genome sequence (Aidley *et al*., in preparation) – to identify all putatively phase-variable loci in *C. jejuni* strain 81-176 through extraction of SSRs, and the functions of their cognate genes, from the genome sequence (GenBank CP000538.1). The presence of PV genes in the genome sequence was also confirmed through a manual search for poly-G/C tracts using the ‘mark from pattern function’ in Artemis. *C. jejuni* strain 81-176 was found to encode 20 putative PV genes, which are shown alongside their *C. jejuni* strain NCTC11168 homologues in Table 1. There were 16 PV genes with a repeat tract within the reading frame and four with intergenic SSRs. The majority (*n*=17) of SSRs were poly-G in the direction of coding, while two were poly-C and one poly-A. For ten of the genes in the *C. jejuni* strain 81-176 phasome, no functional information was obtainable from the annotation. Five of the PV genes (*CJJ81176_1419* to *CJJ81176*_*1435*) are located in the capsular polysaccharide (CPS) locus that encodes several enzymes involved in the transport, biosynthesis or modification of CPS, while four (*CJJ81176_1312* to *CJJ81176_1327*) are in the flagellar O-glycan biosynthesis locus. The other PV genes include a lipo-oligosaccharide (LOS) biosynthesis enzyme (*CJJ81176_1160*), a putative invasion (*CJJ81176_0708*), an amidohydrolase (*CJJ81176_0105*), an anion transporter (*CJJ81176_0086*) and a protein with a putative role in flagellar motility (*CJJ81176_0082*).

**Table 1.** Phase-variable genes in the genome of *C. jejuni* strain 81-176

Mix	Amplicon size (colour)*	Gene (81-176)	11168 homologue (% identity)†	On length‡	SSR (repeats)§	Functional annotation
A	313 (B)	*CJJ81176_0082*	*cj0044c* (95 %)	IG	G (9)	Motility
	353 (B)	*CJJ81176_0590*	n/a	IG	G (10)	Hypothetical
	253 (B)	*CJJ81176_1325*	n/a	9	G (9)	Formyl transferase domain protein
	186 (B)	*CJJ81176_1419*||	*cj1420c* (99 %)	9	G (9)	Putative capsular methyltransferase
	469 (B)	*CJJ81176_1429||*	*cj1429c* (95 %)	9	G (10)	Hypothetical
B	155 (B)	*CJJ81176_0086*	*cj0046*	9	G (9)	Anion transporter
	130 (B)	*CJJ81176_0708*	*cj0685c* (99 %)	9	C (9)	Invasion protein
	224 (B)	*CJJ81176_0758*	*cj0735* (99 %)	10	G (9)	Hypothetical
	288 (B)	*CJJ81176_1160||*	*cj1143*/*cgtA* (66 %)	10	G (10)	beta-1,4-*N*-acetylgalactosamine transferase
	392 (B)	*CJJ81176_1341*	*cj1342c* (75 %)	9	G (9)	Hypothetical
C	167 (G)	*CJJ81176_0646*	*cj0617* (99 %)	10	G (9)	Hypothetical
	161 (Y)	*CJJ81176_1312||*	*cj1295*	9	G (9)	DMGA modification
	291 (G)	*CJJ81176_1321*	*cj1305c* (85 %)	IG	G (9)	Hypothetical
	262 (G)	*CJJ81176_1420||*	*cj1421*	9	G (10)	MeOPN transferase to –4-Galm [22]
	342 (G)	*CJJ81176_1432||*	*cj1139* (61 %)	9	G (9)	Galactosyltransferase
D	287 (Y)	*CJJ81176_0765*	n/a	IG	C (11)	Hypothetical
	197 (Y)	*CJJ81176_0206*	*cj0170* (100 %)	8	G (9)	Hypothetical
	233 (G)	*CJJ81176_1327*	*cj1305c* (71 %)	9	G (9)	Hypothetical
	321 (Y)	*CJJ81176_1435||*	*cj1422*	9	G (9)	MeOPN transferase to 2-Gal and 6-Gal [22]
		*CJJ81176_0105*	*cj0067* (99 %)	10	A (10)	Amidohydrolase family protein

*Numbers indicate the expected amplicon sizes for each primer pair, while brackets indicate the fluorescent colour of each product as detected by PeakScanner (B, blue; G, green; Y, yellow).

†Identity scores are provided where coverage is greater than 60%.

‡IG, intergenic.

§The parentheses contain the number of repeats as determined by Sanger sequencing of a control genomic DNA sample.

||Denotes a gene that is known to be involved in capsular, LOS or flagellin modification.

### High diversity in five phase-variable genes in populations from colonized chickens

To study switches in expression of all putative PV genes of strain 81-176, we developed the 19-locus-CJ81176 PV-analysis assay that allows determination of repeat numbers and expression states for multiple colonies within a population [14, 15, 17]. This assay enabled examination of the PV states for populations of *C. jejuni* strain 81-176 derived from *in vivo* experiments. We applied this assay to birds that were either non-treated or vaccinated with an *E. coli* strain expressing the *C. jejuni* N-glycan as described by Nothaft *et al*. [14].

There were 259 colonies derived from the caecal contents of nine non-vaccinated birds colonized at 7.2×10^9^ to 4.6×10^10^ c.f.u. g^−1^ caecal content, and 410 colonies from 15 (out of 36) vaccinated birds that were still colonized at 2.3×10^8^ to 5.0×10^10^ c.f.u. g^−1^ caecal content. In the remainder of birds (21 out of 36), no colonization was observed. The colonies from both the non-vaccinated and the vaccine ‘non-responder’ birds and inoculum were subject to GeneScan analysis to determine ON/OFF expression states.

**Fig. 2.**
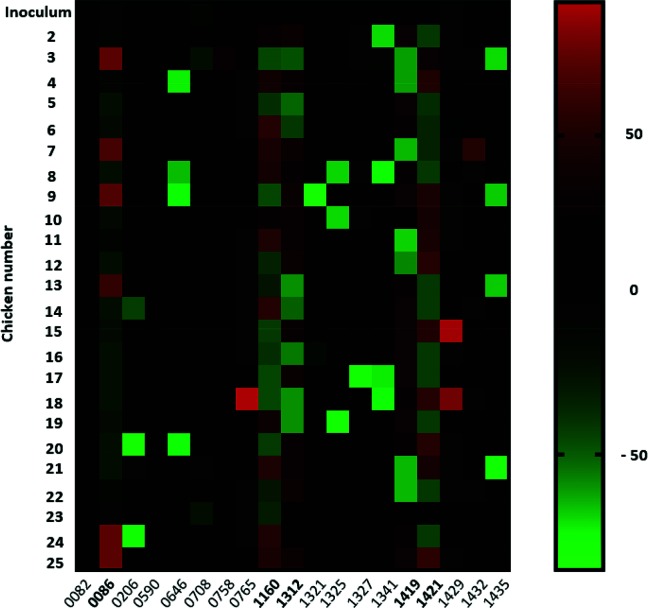
Deviation from the mean percentage ON for output and input populations for 19 PV genes. Three-week old naïve and N-glycan-immunized chickens were orally gavaged with 1×10^6^ c.f.u. of *C. jejuni* strain 81-176. Output populations were recovered at 7 days post challenge from the caeca of 9 naïve and 15 immunised birds. Repeat numbers for 19 phase-variable genes were determined for up to 30 colonies per caecum sample from 24 birds and 30 colonies for the input (inoculum) population. Mean ON percentages were calculated based on the combined data from all chickens. The deviation from this mean was calculated for each gene and sample and plotted on a heat map. Red, >50 % more than the mean % ON; green, >50 % less than the mean % ON; black, identical to the mean % ON. Numbers on the *y*-axis refer to specific chickens. The ‘*CJJ81176*’ locus tag is omitted from the gene names on the *x*-axis.

We previously reported the overall percentage ON state for each gene in each colonized bird and demonstrated, using hierarchical clustering, that control and immunized birds exhibited similar expression profiles [14]. We also reported that a sub-set of genes exhibited the highest levels of bird-to-bird variation. In this article, we describe a more detailed examination of bird-to-bird variation and the distribution of phasotypes (combinatorial expression states). Firstly, we calculated the mean ON/OFF value for each gene across all chickens and plotted the deviation in %ON from this mean, for each gene and each chicken (Fig. 2). For all but five genes, deviation from the mean %ON was minimal with only occasional exceptions such as chickens 4, 9 and 20, where gene *CJJ81176_0646* showed ~60 % deviation from the mean %ON. Five genes (*CJJ81176_0086*, *CJJ81176_1160*, *CJJ81176_1312*, *CJJ81176_1419* and *CJJ81176_1421*) displayed high levels of variation with six or more chickens showing a large deviation (50–70 %) from the average %ON value. These five genes had a distribution of colonies in both the ON and OFF state in the inoculum sample with %ON states of >10 % and <90 %, indicating that both expression states were present in significant amounts in the inoculum (data not shown). Only two other genes (*CJJ81176_0708* and *CJJ81176_1341*) exhibited %ON states within this range, whereas other genes were mainly in an ON or OFF state.

### Tract lengths of genes in the founder population do not account for high levels of variability at the endpoint

**Fig. 3.**
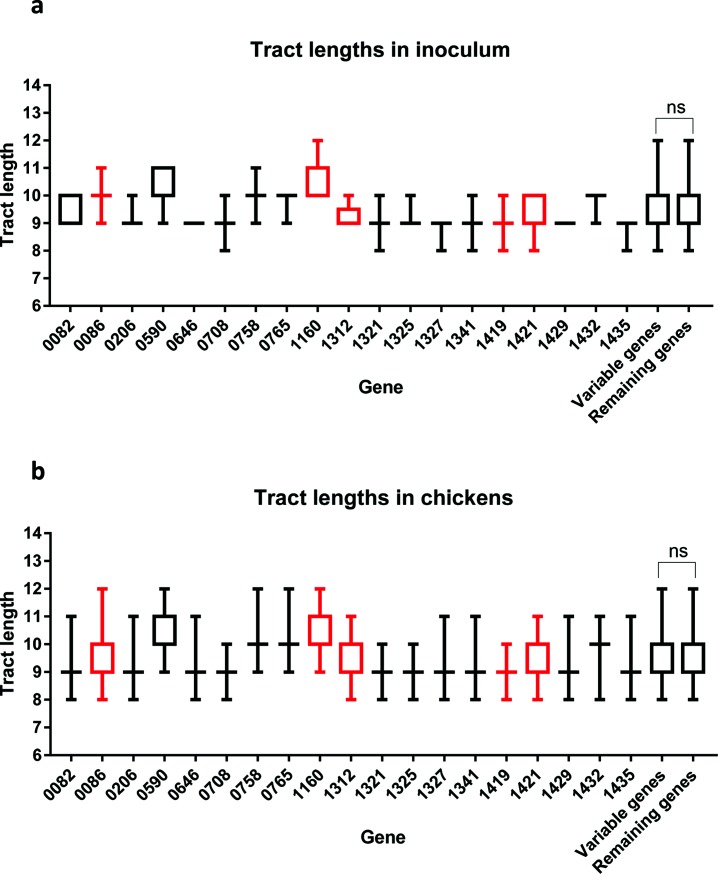
Mean and range of repeat numbers for all PV genes. Repeat numbers for the inoculum sample were extracted from the output data produced by PSAnalyse. Graphs indicate the mean repeat number(s) as a line or box and the range of repeat numbers as error bars. Panel (a), inoculum. Panel (b), combined output populations from 24 chickens. Values highlighted in red reprsent phase-variable genes determined as exhibiting high variability in %ON states in output populations (see text). Values for the repeat numbers for the five most variable genes were combined and compared to repeat number values for the remaining genes. Statistical analyses were performed using an unpaired *t*-test (ns, not significant). The ‘*CJJ81176*’ locus tag is omitted from the gene names on the *x*-axis.

High levels of variation may be a function of tract length or a wide range of tracts present in the inoculum population. Using output data from PSAnalyse [15], mean tract lengths were plotted for all PV genes for each chicken sample and for the inoculum (Fig. 3a). Two genes of the inoculum (*CJJ81176_0646* and *CJJ81176_1429*) encoded G tracts of nine repeats, but with no variation in tract length. Both of these genes displayed low variation in the output populations (Fig. 2). The remaining genes encoded intermediate tract lengths (8–11 repeats), and up to three repeat-unit length heterogeneity. The five most variable genes in the output population (*CJJ81176_0086*, *CJJ81176_1160*, *CJJ81176_1312*, *CJJ81176_1419* and *CJJ81176_1421*) fell into this latter group with between 2 and 3 repeat-length heterogeneity in the inoculum. Overall, no significant difference was detected between the tract lengths in the inoculum for the five most variable genes and all the other phase-variable genes (*P*=0.9976; unpaired *t*-test comparing these two gene groups) (Fig. 3a).

We then pooled together the tract lengths observed for each gene, in all chickens, to determine the spread of repeat tract lengths occurring during colonization. Interestingly, a spread of repeat units was observed across all genes (Fig. 3b), indicating a rapid development of repeat tract diversity following colonization. When a similar analysis was performed with all repeat tract lengths observed in individual chickens, a spread of repeat numbers was also observed, indicating either rapid development of repeat tract diversity, or colonization by multiple bacteria with differing repeat tract profiles (data not shown).

### Following mixed inoculation, broiler chickens are colonized by *C. jejuni* populations dominated by a single phasotype

**Fig. 4.**
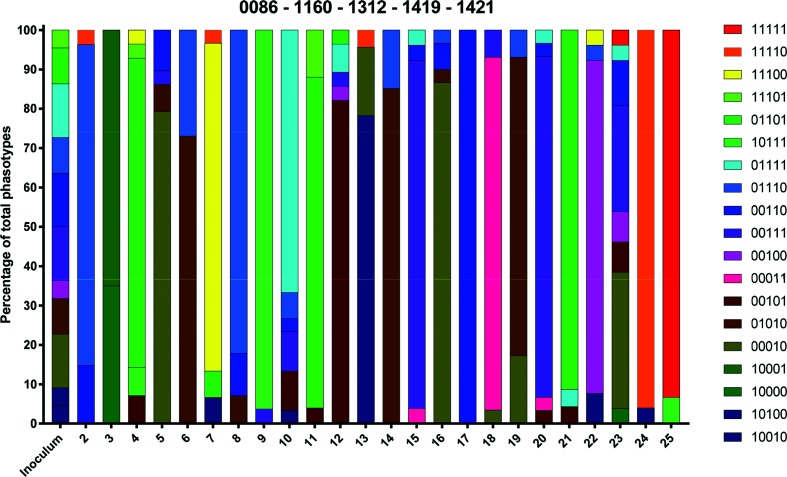
Phasotypes for the five most variable genes in individual bird samples. Binary ON/OFF data for each gene were coded as 1 for ON and 0 for OFF. Binary values were combined for the five most variable genes to generate a five gene phasotype with the following order: *CJJ81176_0086*, *CJJ81176_1160*, *CJJ81176_1312*, *CJJ81176_1419* and *CJJ81176_1421*. Frequencies were determined from analysis of up to 30 colonies derived from the inoculum and each bird sample.

Repeat tract lengths were converted into expression states enabling analysis of combinatorial expression states (phasotypes). In order to facilitate analysis, the genes were split into three 5- and one 4-gene phasotype. A variable 5-gene phasotype was composed of the 5 most variable genes. Fig. 4 shows the phasotype diversity for the variable 5-gene phasotype in the inoculum and the populations arising from each individual chicken. The total number of possible phasotypes for these 5 genes is 32, and 19 were observed across the 720 colonies from these experimental populations. The inoculum was mixed and contained 11 phasotypes. In contrast, each chicken (with the exception of chicken 23) was colonized by a single dominant phasotype (>60 % of the total number). Unexpectedly, several chickens were colonized by a dominant phasotype that was not detected in the inoculum (11100, chicken 7; 10111, chicken 9; 00101, chicken 12; 00011, chicken 18; 11110, chicken 24; and 11111, chicken 25). However, several of these missing phasotypes were just a single gene switch away from a phasotype present in the inoculum (for example, inoculum phasotype 11101 could have undergone an ON-to-OFF switch in *cjj81176-1421* to generate output phasotype 11100), indicating either that early switching events may give rise to non-inoculum phasotypes dominating the chickens, or that these phasotypes were below the detection threshold of 0.033 (i.e. less than one colony for the 30 analysed colonies of the inoculum).

For the other two five gene phasotypes, 12 and 14 of a possible 32 phasotypes were observed (Figs S1 and S2) while for the 4-gene phasotype, 10 of a possible 16 phasotypes were detected (Fig. S3). In all cases, the inoculum had a smaller number of phasotypes for these gene groups than that observed for the variable 5-gene phasotype and tended to be dominated by one major phasotype. As before, dominance of a single phasotype was readily observed in each of the chicken output populations, with this dominant type usually being the major inoculum phasotype. However, between five and seven chickens for each of these gene combinations contained one of the minor inoculum phasotypes or a close derivative thereof.

### The number of chickens colonized with a given phasotype correlates with composition of the inoculum

**Fig. 5.**
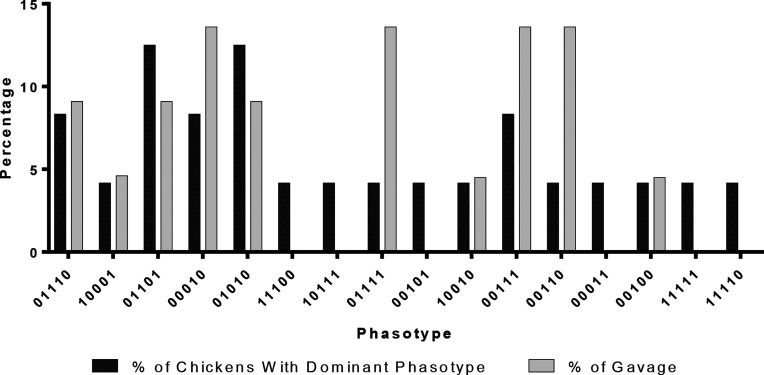
Comparison of dominant phasotype frequencies for *in vivo* output populations with prevalence in the input population. The dominant phasotype in each output population (i.e. individual bird caecal sample) was defined as the phasotype present in >60 % of colonies. The number of birds with a specific dominant phasotype was divided by the total numbers of birds (*n*=24) and converted into a percentage (black bars). The frequency of each dominant phasotype in the inoculum was determined by dividing the number of colonies with a specific phasotype by the total number of colonies (*n*=30) and converting into a percentage (grey bars).

To investigate colonization efficiency, and/or random sorting of the inoculated population, we determined the percentage of chickens colonized by a dominant phasotype and the percentage composition of the inoculum. A dominant phasotype was defined as the phasotype comprising 60 % or more of colonies derived from a specific bird, and was assessed for all samples using the variable 5-gene phasotype. Apart from bird 23, all birds were colonized by a dominant phasotype and, in 13 of 19 cases, the output phasotype was detected in the inoculum (Fig. 5). In all cases where a phasotype was present in the inoculum, this phasotype was sorted into an equivalent percentage of chickens. For example, the 01110 phasotype is present in 8 % of the colonies derived from the inoculum and is dominant in 7 % of chickens. Three phasotypes (01111, 00111 and 00110) were present with an equal percentage in the inoculum (14 % each, 42 % in total), but dominantly colonized a lower number of chickens in all cases (4, 7 and 4 % respectively). Finally, six phasotypes (11100, 10111, 00101, 00011, 11111 and 11110) dominantly colonized 4 % of chickens but could not be detected in the inoculum. These data indicate that the reduction in diversity observed in each chicken is likely *non-*selective, as no phasotype had preferentially colonized with a disproportionate representation between the inoculated and output populations.

### Comparison with simulated diversity and divergence data indicates a single-cell bottleneck in colonization of chickens

**Fig. 6.**
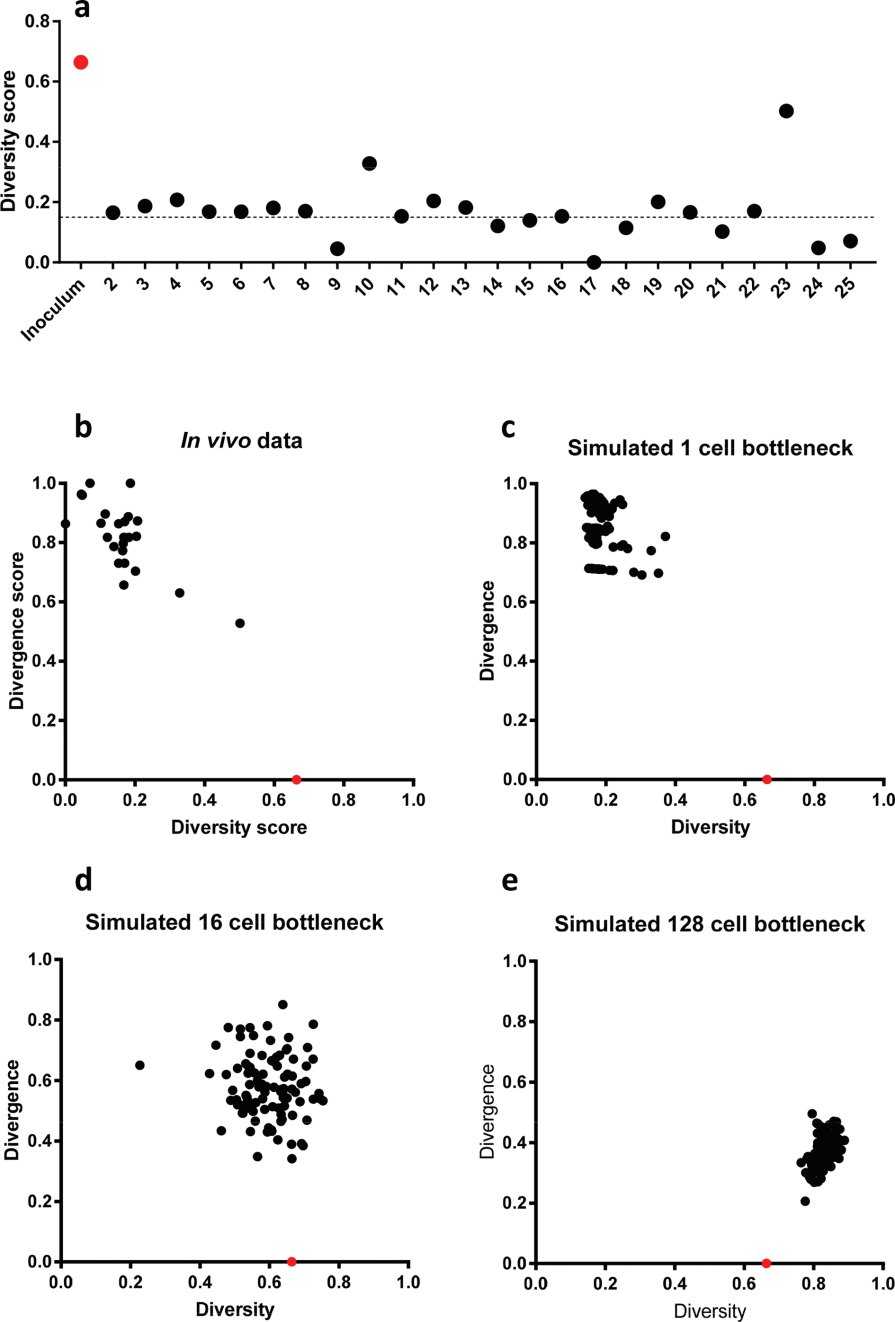
Diversity and divergence scores for the variable 5-gene phasotypes in input and *in vivo* output populations. Diversity scores were calculated using the numbers of colonies with each phasotype for the phasotype composed of the five most variable genes (see Fig. 4). A score of 1 is the highest possible value for diversity, whereby all phasotypes are present in equal proportions while 0 is the lowest diversity score, whereby a single phasotype is present in all analysed colonies. Divergence from the input population was determined using a population separation equation. A divergence score of 1 indicates that there are no phasotypes in common with the inoculated population, whereas a divergence score of 0 indicates that the phasotype composition of the sample is identical to the inoculum. Panel a, diversity scores for all input and output populations from the *in vivo* experiment with 24 birds. The dotted line is the mean diversity score for all chickens. Panel b, a plot of diversity versus divergence for all output population. Panels (c–e), *in silico* simulations were performed using a previously published model to determine the effects of bottleneck size on phasotype prevalence in *C. jejuni* populations [11]. The input for the model was the phasotype distribution obtained from the inoculum of the *in vivo* dataset. The model was run for a single bottleneck with sizes of 1 (c), 16 (d) and 128 (e) cells. The diversity of the input (inoculum) population is shown by a red point on each graph. Numbers on the *x*-axis of panel (a) refer to specific chickens.

In order to examine the size of the population bottleneck, we performed an *in silico* simulation using a previously published method [11]. Scores for sample diversity and divergence from the inoculum were calculated for the variable 5-gene phasotype. Fig. 6(a) indicates that high amounts of diversity were present in the inoculated population (diversity score; 0.664), whereas 23 of 24 birds had reduced diversity scores ranging from 0 to 0.4. Chicken 23 showed a higher diversity score than the other chickens (diversity score; 0.5), but this was still reduced in comparison to the inoculum. We then plotted divergence scores against diversity scores for each respective chicken. Fig. 6(b) indicates that all chickens cluster towards having high levels of divergence from the inoculum (divergence scores between 0.5 and 1.0), and low levels of diversity. We then performed an *in silico* simulation of changes in the *C. jejuni* population using the observed inoculum phasotype composition and assuming one bottleneck with a size of 1, 16 or 128 cells (Fig. 6). A close qualitative match was observed between the experimental populations and the simulated populations with a single-cell bottleneck. A non-parametric permutational multivariate analysis of variance test revealed that output populations from the *in vivo* dataset were not significantly different from those of the *in silico* single-cell bottleneck populations (*P*-value of 0.522), whereas the differences between the *in vivo* dataset and the 16-, and 128-cell bottleneck simulations were significant (*P* values of <0.001). Simulations were also run with three to five sequential bottlenecks. Only minor differences were observed between the *in silico* models of one, three and five bottlenecks (Figs 6, S4 and S5, respectively), indicating that one single-cell bottleneck could have given rise to the experimentally observed population structure.

## Discussion

Maintenance of a hypermutable SSR in a phase-variable gene is driven by alternating selection for the fitness benefits of two opposing expression states such as the ON and OFF states of translational PV. Thus *C. jejuni* is likely to require its whole repertoire of PV genes with selection for these loci driven by the niches associated with environmental transmission, colonization of poultry, persistence in the face of innate and adaptive human immune responses [12, 13, 18–20], competition from other microbes and frequent predation by bacteriophages [21]. While selection is a key driver of evolution of these loci, other population determinants such as bottlenecks and hitch-hiking will influence the population structure of phase variants during short-term infections. Studying the whole repertoire of PV genes in a given strain *in vivo* is likely to yield insights into host adaptation and fluctuations in population structure. Lango-Scholey *et al*. [15] described the application of a high-throughput multiplex PCR/GeneScan assay to analysis of PV in experimental populations of *C. jejuni* strain NCTC11168. Nothaft *et al*. [14] described the application of a similar assay for analysis of *C. jejuni* strain 81-176 populations obtained from chickens. In this case [14], similar hierarchical patterns were observed for the %ON states of *C. jejuni* populations colonizing naive birds versus birds given the *C. jejuni* N-glycan vaccine. In this manuscript, we describe the further examination of these populations for changes in repeat number (a determinant of mutability) and the combinatorial expression states of phase-variable genes, leading to identification of a severe population bottleneck during colonization of birds.

Hierarchical clustering of %ON states identified five genes as exhibiting differential patterns and higher variability than other phase-variable genes [14]. The variability of all phase-variable genes was re-examined by plotting the deviation in %ON observed for each bird from the average %ON calculated for all birds. The same five genes deviated more frequently than other genes from the inoculum population in output populations (Fig. 2). Thus *CJJ81176_0086* had %ON values in six birds that were >50 % higher than the mean value. For the other four variable genes, individual birds generally had values of >50 % less than the mean. The *CJJ81176_1421* gene is homologous to *cj1421* and *cj1422*, which encode transferases responsible for addition of O-methyl phosphoramidate (MeOPN) to the CPS of *C. jejuni* strain NCTC11168 [22]. In strain 81–176, *CJJ81176_1421* transfers MeOPN to the C-4 position of Gal [23]. The MeOPN modification contributes to CPS-mediated serum resistance and hence may be a virulence factor with a key role in resisting attack from the innate immune response in the chicken and human gut [20, 23, 24]. Studies have also demonstrated that the MeOPN modification is a receptor for bacteriophage infection in *C. jejuni* strain NCTC11168 [25], and that lytic phages select for OFF expression states of *cj1421* both *in vitro* and *in vivo* [25–27]. Interestingly, *CJJ81176_1419* encodes a homologue of Cj1420, a putative methyltransferase involved in MeOPN biosynthesis in *C. jejuni* NCTC11168 that could also influence both serum and phage sensitivity (Wenzel *et al*., in preparation). Extreme variations in %ON values for these genes between birds may be due to differential exposure to the host immune response (selecting for ON states) or phage (selecting for OFF states). The other variable genes code for an anion transporter (pseudogene; *CJJ81176_0086*), an LOS beta-1,4-N-aminogalactosaminyltransferase (*CJJ81176_1160*; known to be involved in ganglioside mimicry [18]) and an enzyme within the flagella glycosylation locus (*CJJ81176_1312*). A homologue of the latter protein was demonstrated to be involved in addition of dimethylglyceric acid (DMGA) to the O-linked pseudaminic acid residues on flagella of *C. jejuni* NCTC11168 [28]. Explanations for bird-to-bird variability in selection for differential expression states of these genes could therefore be due to differences in phage composition, immune recognition, or microbial populations in the chickens.

Alternative explanations for the high variability in these five genes, as compared to the other phase-variable genes, include (1) that there was a higher variability in tract lengths in the inoculum and (2) a tendency for longer tracts. The association between mutability and repeat number was observed when a tenfold higher PV rate was detected for a G11 tract as compared to a G8 tract in a *cj1139-lacZ* reporter gene, also encoding an enzyme involved in PV of LOS ganglioside mimics in *C. jejuni* strain NCTC11168 [7]. However, no significant differences were detected between the range or number of repeats of the highly variable genes as compared to all other genes in this study (Fig. 3). Thus, variability in output populations was not due to pre-existing variation or differences in mutability.

Non-selective or selective bottlenecks are known to constrain genetic variability and to introduce stochastic effects on genetic population structure. Bacterial populations are frequently subject to movement between environmental niches or compartments within the host (e.g. nasopharynx into bloodstream). These translocations are known to impose bottlenecks on bacterial populations [8, 9], and subsequently, the rapid generation of phenotypic diversity through PV has the potential to mitigate the restrictive effects of bottlenecks. Detection of bottlenecks relies on having a genetically diverse inoculum and an ability to identify variants in the output population. Previous studies in bacteria have relied on isogenic strains marked with antibiotic resistan﻿﻿ce cassettes [29] or short, variable sequences while in human populations, micro-satellites provide one measure of bottlenecks [30]. Multi-gene phasotypes based on SSRs in repeat-mediated phase-variable genes provide a basis for detecting and tracking specific genotypes. While each individual PV state is subject to high mutability in the SSR, transitions between phasotypes are rarer due to the multiplicative effects of mutation, so that if the PV rate is 1×10^3^ for each gene then a simultaneous change﻿ in two genes is 1×10^6^.

Examination of the phasotype distributions for a five gene phasotype of the most variable genes provided evidence of a major bottleneck. The inoculum fortuitously contained similar numbers of multiple phasotypes. These phasotypes were distributed in a random pattern across the 24 birds. Most birds were dominated by a single phasotype, providing a further indication of a narrow bottleneck. Similar patterns were observed for the other gene phasotypes but with lower bird-to-bird variation due to limited differences in the inoculum. Aidley *et al*. [11] described a simple *in silico* model for simulation of the effects of non-selective bottlenecks on phasotype distributions in *C. jejuni* populations [11]. This simulator utilizes experimentally determined *C. jejuni* PV rates, but assumes that all genes switch at the same rates and that ON-to-OFF and OFF-to-ON rates are identical. PV rates were determined for reporter and native genes in *C. jejuni* strain NCTC11168, but are likely to be representative of PV rates in all *C. jejuni* strains. Another key aspect of the model is the number of generations between bottlenecks and between input and output populations, as PV states of multiple genes are expected to coalesce to a steady state after multiple generations in the absence of selection [7]. Simulation of the effects of different-size bottlenecks on the *in vivo* distribution of the five-gene variable phasotype of *C. jejuni* strain 81-176 indicated that the strongest quantitative match was to the pattern produced by single-cell bottlenecks, and indeed matched the pattern produced by one single-cell bottleneck.

This study indicates that non-selective single-cell bottlenecks may be a key feature of the *Campylobacter* life cycle during natural infections. Narrow bottlenecks were previously intimated by the failure of signature-tagged mutagenesis schemes in *C. jejuni* and confirmed by performance of mixed infections with wild-type isogenic marked strains [31]. In both cases, there was a failure to recover multiple independent isolates from individual birds. The present experiment successfully detected a bottleneck due to use of a small, highly mixed inoculum and individual housing of birds. In most other *Campylobacter* experiments and in broiler flocks, there is a strong potential for constant challenge of birds, due to frequent consumption of *Campylobacter*-containing faecal material. This results in loss of bird-to-bird variation as adaptive variants can spread between birds and obscure evidence of non-selective bottlenecks. Thus, the relevance of bottlenecks to spread within actual chicken flocks requires further exploration to determine whether it is a major or minor determinant of phenotypic variability and the rate of adaptation. This is particularly important as broiler chickens are the primary source of *Campylobacter* infections in humans [32], and it is unclear whether specific phasotypes have a higher propensity to cause disease in humans or are more likely to cause invasive disease in chickens and hence may transmit more frequently through the food chain [33].

Another important question is how transmission from the oesophagus/crop of chickens to the caecum imposes bottlenecks on *Campylobacter*. The most parsimonious explanation of our results is that an arbitrary, non-selective reduction in cell number occurred as the *C. jejuni* population passed through the gastrointestinal tract so that only a small number of cells reached a site permissive for replication. This small number of individual cells then initiated formation of micro-colonies, of which one outcompeted the others to generate a clonal, persistent population. An alternative is that the caecum is a privileged site permissive for colonization by only certain genotypes of *C. jejuni*. In this case, we would speculate that this permissive genotype was generated infrequently during initial replication of *C. jejuni*, resulting in stochastic association with different phasotypes and hence resulting in a narrow selective bottleneck that was linked to, but not dependent on, PV states. Factors crucial for colonization of chickens by *C. jejuni* have been identified [33, 34], but future studies to identify the source of within-host bottlenecks are required to define the key mechanisms and adaptive strategies utilized for chicken colonization by *C. jejuni*.

In conclusion, we show that random sorting of phasotypes between individuals and generation of clonal populations occurred when chickens were inoculated with a mixed *C. jejuni* population. Conversely, we did not detect amplification of a specific phasotype, indicating that selection for specific phasotypes does not occur when this *C. jejuni* strain colonizes chickens. By comparison to simulated data, we conclude that *C. jejuni* populations are subject to single-cell bottlenecks during colonization of chickens. The high degree of genetic heterogeneity imposed by such severe bottlenecks during host colonization may have implications for understanding the frequency of food contamination and outcomes on human disease by this important pathogen.

## Supplementary Data

204 Supplementary File 1
