# Supplementary material for: Random sorting of Campylobacter jejuni phase variants due to a narrow bottleneck during colonization of broiler chickens
Source: Microbiology (Reading). 2018 Jun 1;164(6):896–907. doi: 10.1099/mic.0.000669 (PMC6097035; doi:10.1099/mic.0.000669)
Supplement: Supplementary File 1 [file mic-164-896-s001.pdf]

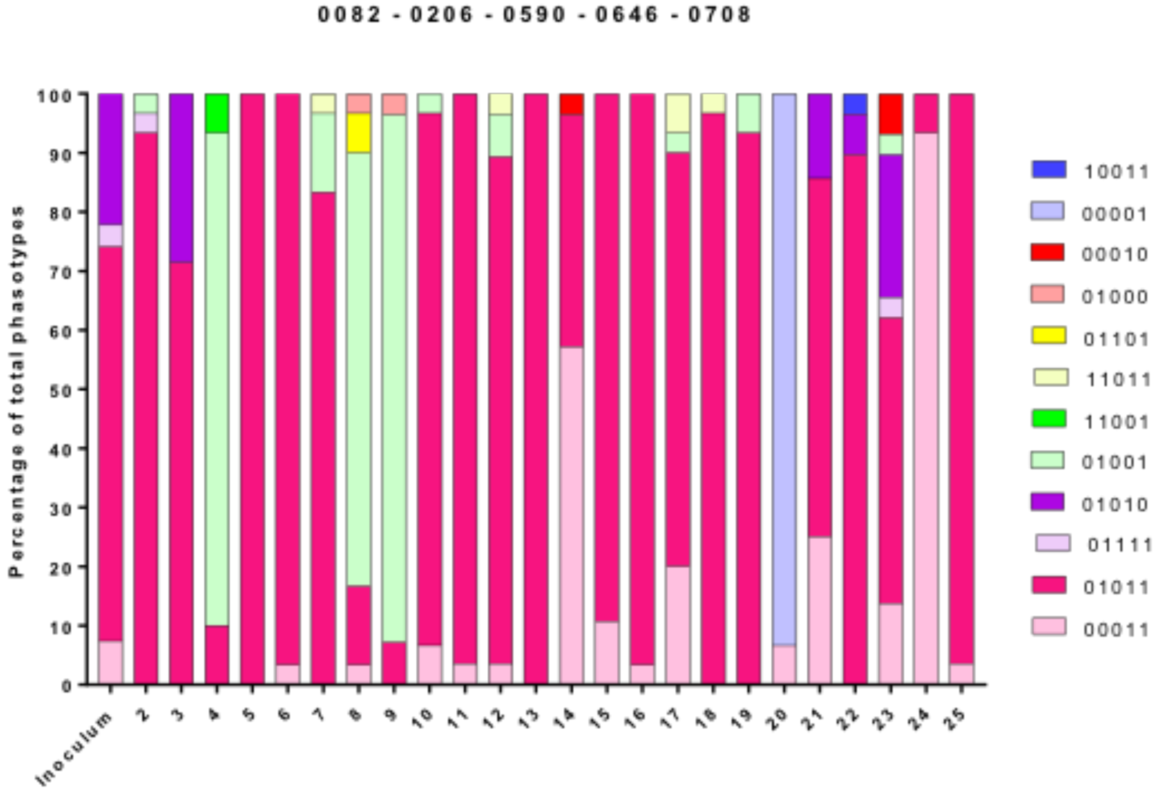

Supplementary Figure 1. Phasotype frequencies for five phase-variable genes (group 2) in all chickens and the gavage. Binary ON/OFF data taken from PSAnalyse are coded 1 for ON, and 0 for OFF. The legend shows the order of the five variable genes coded; *CJJ81176_0082*-*CJJ81176_0206*-*CJJ81176_0590*-*CJJ81176_0646*-*CJJ81176_0708*. The size of the coloured bars indicates the % of the analysed population made up by that phasotype. Numbers on the x axis refer to specific chickens. The '*CJJ81176*' locus tag is omitted from the figure.

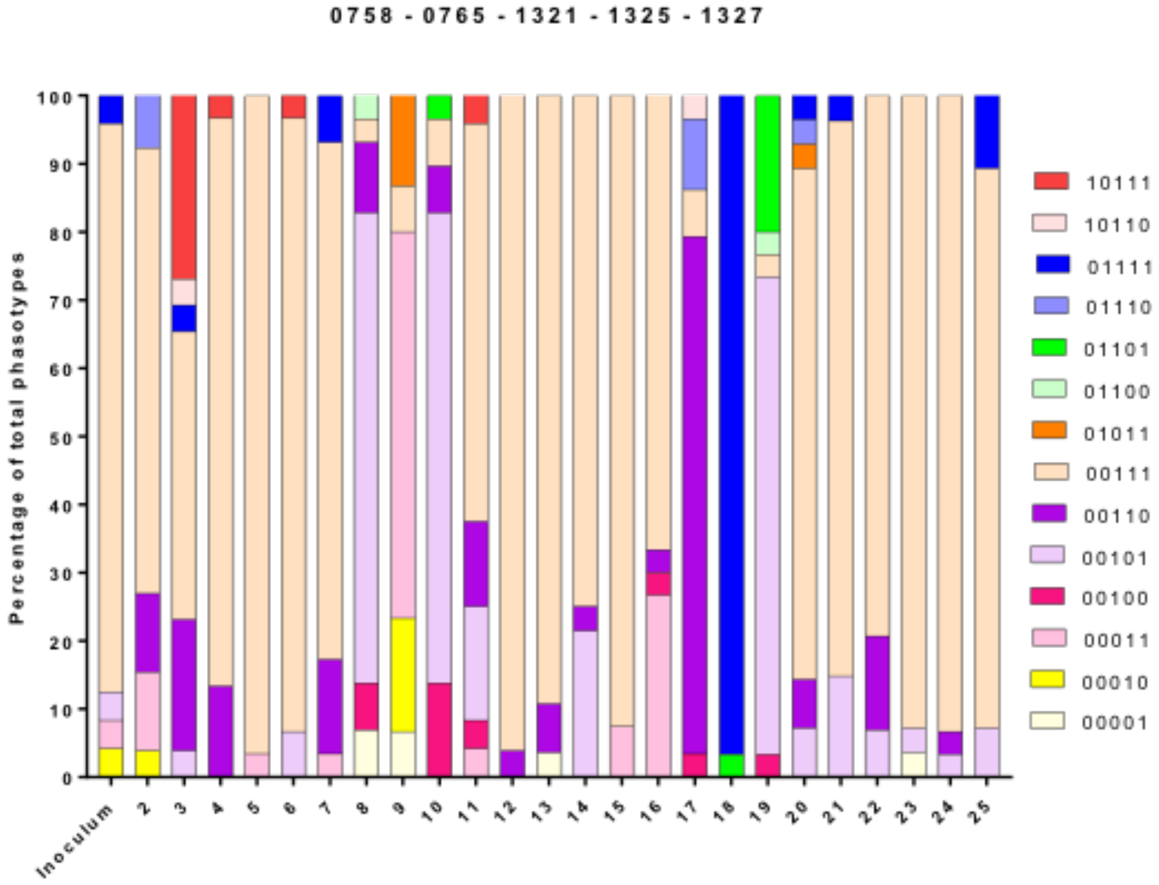

Supplementary Figure 2. Phasotype frequencies for five phase-variable genes (group 3) in all chickens and the gavage. Binary ON/OFF data taken from PSAnalyse are coded 1 for ON, and 0 for OFF. The legend shows the order of the five variable genes coded; *CJJ81176_0758*-*CJJ81176_0765*-*CJJ81176_1321*-*CJJ81176_1325*-*CJJ81176_1327*. The size of the coloured bars indicates the % of the analysed population made up by that phasotype. Numbers on the x axis refer to specific chickens. The '*CJJ81176*' locus tag is omitted from the figure.

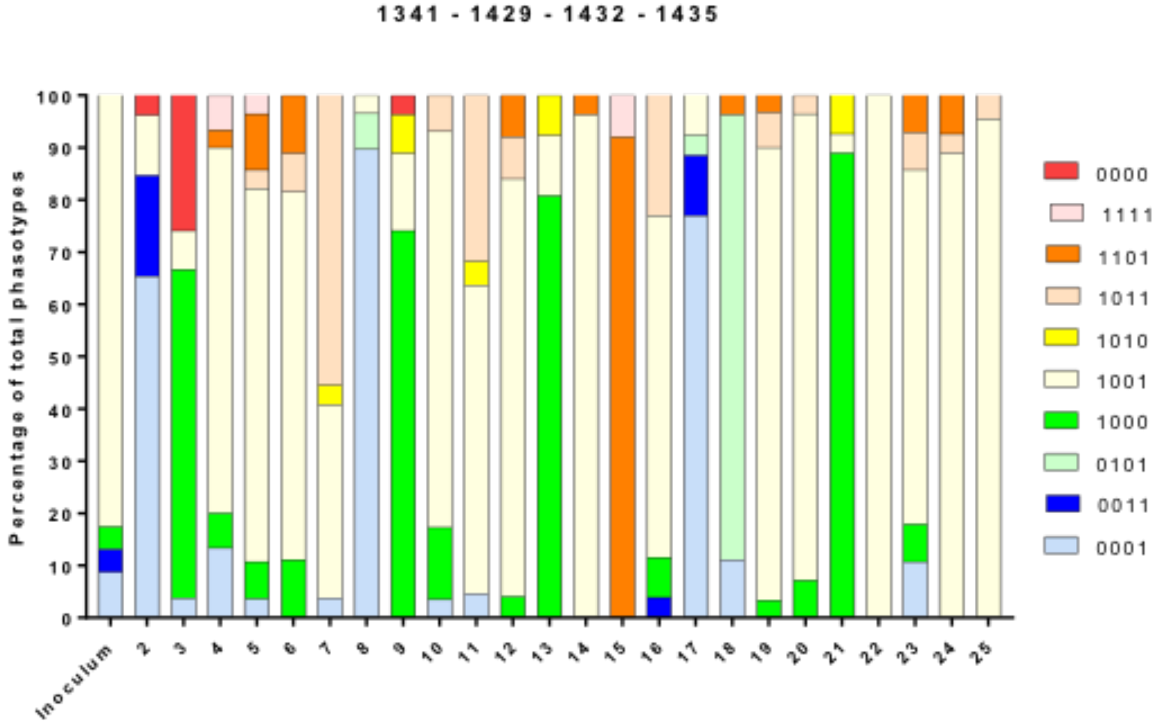

Supplementary Figure 3. Phasotype frequencies for four phase-variable genes (group 4) in all chickens and the gavage. Binary ON/OFF data taken from PSAnalyse are coded 1 for ON, and 0 for OFF. The legend shows the order of the four variable genes coded; *CJJ81176_1341-CJJ81176_1429-CJJ81176_1432-CJJ81176_1435*. The size of the coloured bars indicates the % of the analysed population made up by that phasotype. Numbers on the x axis refer to specific chickens. The '*CJJ81176*' locus tag is omitted from the figure.

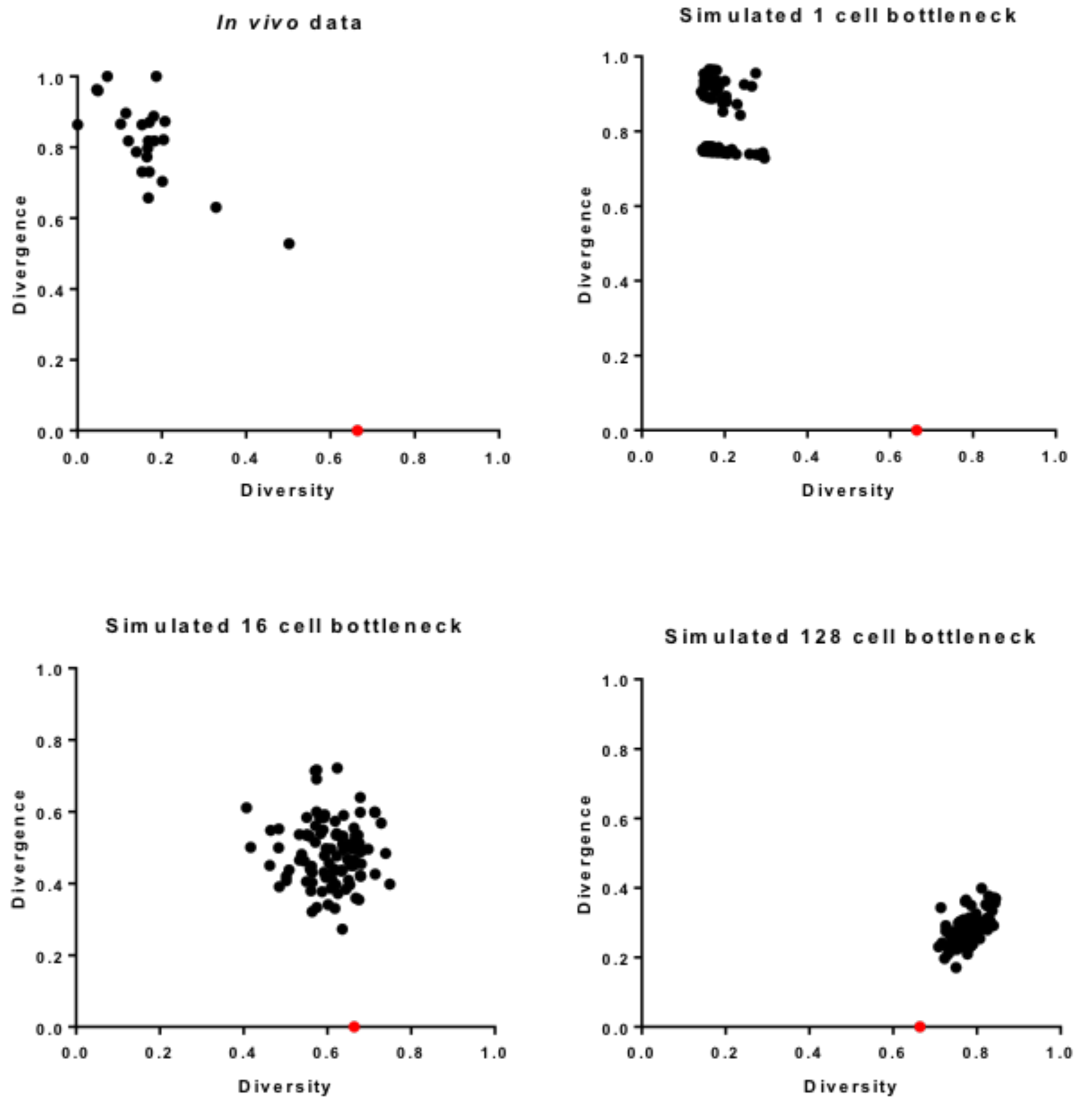

Supplementary Figure 4
Diversity scores were calculated using the numbers of colonies with each phasotype for the phasotype composed of the five most variable genes (see Figure 5). *In silico* simulations were performed using a previously published model to determine the effects of bottleneck size on phasotype prevalence in *C. jejuni* populations [11]. The input for the model was the phasotype distribution obtained from the inoculum of the *in vivo* dataset (*in vivo* data panel). The model was run for THREE consecutive bottlenecks with sizes of 1, 16, and 128 cells. The diversity of the input (inoculum) population is shown by a red point on each graph. Numbers on the x axis refer to specific chickens.

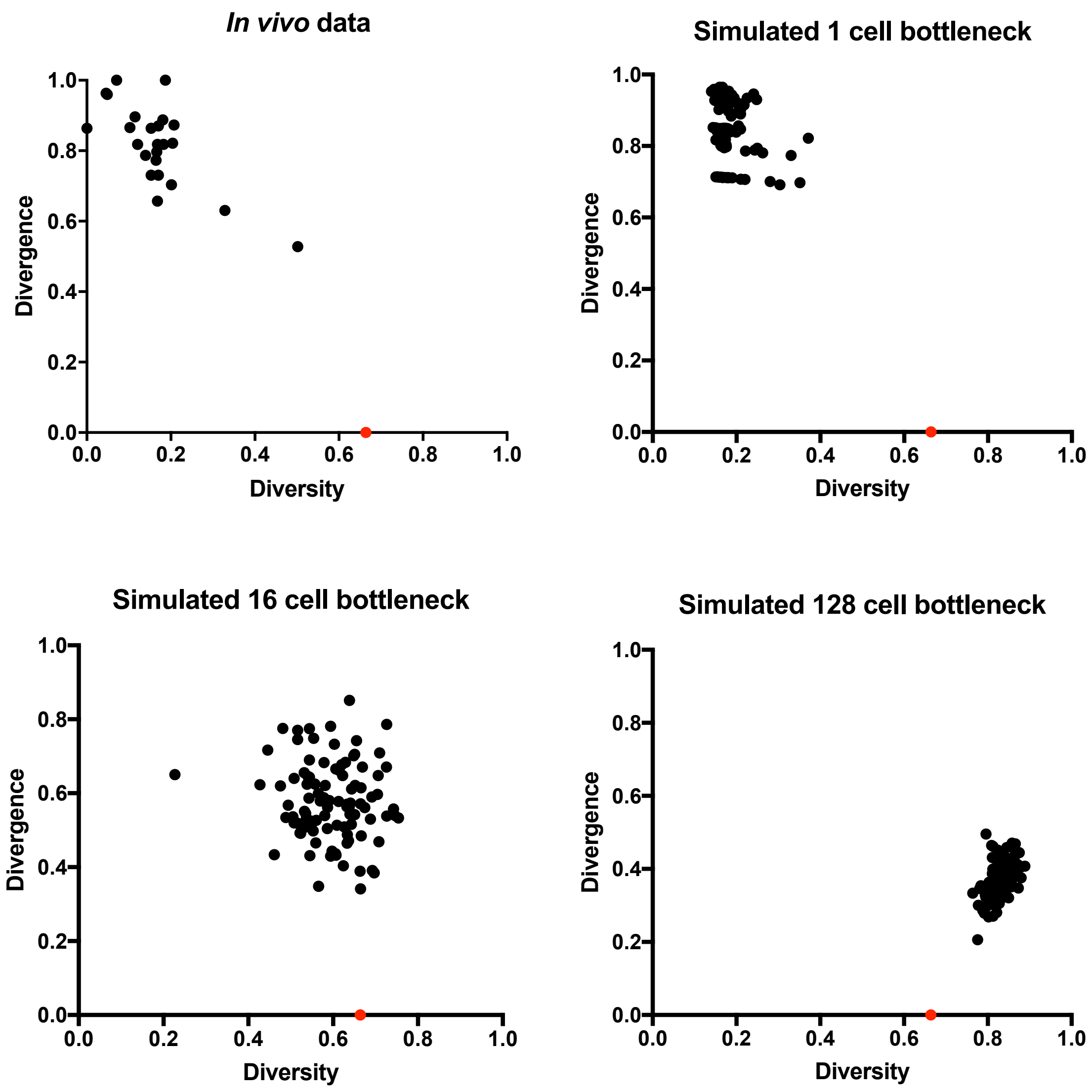

Supplementary Figure 5
Diversity scores were calculated using the numbers of colonies with each phasotype for the phasotype composed of the five most variable genes (see Figure 5). *In silico* simulations were performed using a previously published model to determine the effects of bottleneck size on phasotype prevalence in *C. jejuni* populations [11]. The input for the model was the phasotype distribution obtained from the inoculum of the *in vivo* dataset (*in vivo* data panel). The model was run for FIVE consecutive bottlenecks with sizes of 1, 16, and 128 cells. The diversity of the input (inoculum) population is shown by a red point on each graph. Numbers on the x axis refer to specific chickens.
